# A Coupled Piezoelectric Sensor for MMG-Based Human-Machine Interfaces

**DOI:** 10.3390/s21248380

**Published:** 2021-12-15

**Authors:** Mateusz Szumilas, Michał Władziński, Krzysztof Wildner

**Affiliations:** Warsaw University of Technology, Faculty of Mechatronics, Institute of Metrology and Biomedical Engineering, A. Boboli 8 St., 02-525 Warsaw, Poland; michal.wladzinski@pw.edu.pl (M.W.); krzysztof.wildner@pw.edu.pl (K.W.)

**Keywords:** mechanomyography, piezoelectric sensor, vibration sensor, human-machine interface, prosthetic control, hand gesture recognition, convolutional neural network

## Abstract

Mechanomyography (MMG) is a technique of recording muscles activity that may be considered a suitable choice for human–machine interfaces (HMI). The design of sensors used for MMG and their spatial distribution are among the deciding factors behind their successful implementation to HMI. We present a new design of a MMG sensor, which consists of two coupled piezoelectric discs in a single housing. The sensor’s functionality was verified in two experimental setups related to typical MMG applications: an estimation of the force/MMG relationship under static conditions and a neural network-based gesture classification. The results showed exponential relationships between acquired MMG and exerted force (for up to 60% of the maximal voluntary contraction) alongside good classification accuracy (94.3%) of eight hand motions based on MMG from a single-site acquisition at the forearm. The simplification of the MMG-based HMI interface in terms of spatial arrangement is rendered possible with the designed sensor.

## 1. Introduction

Mechanomyography (MMG) is a measurement technique used to record muscles activity based on vibrations arising as an effect of muscle fibers mechanical contractions [1,2,3]. This technique is still less popular, especially in clinical applications, compared to electromyography (EMG). Nevertheless, the area of application of mechanomyography is relatively wide [1,2,4]: from human-machine interfaces (HMI), especially prosthetic devices control [5,6] and gesture recognition [7,8,9], to investigations of physiological principles of neuro-muscular system functions [10,11,12]. MMG does not require an electrical connection to the skin, therefore it may be applied without prior skin preparation in unconditioned environments, and the provided response exhibits low variability over time regarding the skin condition, as long as the sensor position is not adjusted [13,14]. Differences between the EMG and MMG signals may be observed, e.g., in their fatigue- and force-related responses, with some examples of MMG showing greater sensitivity than EMG when changes in muscle activation strategies are examined [15,16]. The determinants of proper MMG implementation are actively investigated, among others, in order to eliminate crosstalk from neighboring muscles, improve repeatability and signal-to-noise ratio of acquired signals [17,18,19].

Mechanical vibrations originating from muscle activity can be converted to electrical signal via various types of transducers: accelerometers, microphones, or laser distance sensors [2,14,20]. The main differences are in the methods of ensuring a stable coupling between the sensor and the muscle, which directly affect the achieved frequency response of the MMG setup. Accelerometers are often used for MMG recording due to their low mass, small dimensions, and availability of sensors with integrated signal conditioning and digital output. Among microphones, either contact or non-contact ones might be used. For low-cost design, piezoelectric discs in the role of contact microphones sometimes are the choice because of their wide availability, good sensitivity, and very low price. They might be placed directly on the surface of the skin, above the muscle of interest [21,22]. However, with minimal effort, the way they are used can be modified to obtain signals several times higher in amplitude. Piezoelectric elements may be used in a way to benefit from their strong bending, such as in the case of diaphragm piezoelectric microphones or flexural mode accelerometers. Commercial contact microphones based on such an approach have also been used to record mechanomyographic signals previously [2,23,24].

When the myographic signals are to be used for HMI, one of the essential areas of optimization is the number and placement of the sensors used. The interface must be reliable and intuitive but cost-effective at the same time [25]. This enforces endeavours to improve sensing quality, looking for multimodal approaches and new sensors configurations as well. Coupling sensors together is one possible approach, e.g., Silva and Chau [26] coupled microphone and accelerometer, within one sensor, for effective noise reduction in MMG recording; Gregori et al. developed a combined EMG/MMG sensor, which provided improvement, especially in artifact rejection [27]; similarly, Fukuhara and Oka [28] and Wolczowski et al. [5] used a hybrid EMG/MMG sensor. Zhang et al. [29] showed that an introduction of MMG signals might significantly improve the performance of an EMG-pattern recognition-based prosthetic control.

Here, we present a new MMG sensor design, which consists of two coupled piezoelectric discs in a single housing so that two complementary signals related to the muscle activity may be measured simultaneously in a single site. Such an approach brings simplification of the MMG interface in terms of transducers’ spatial distribution.

The sensor functionality has been verified in two typical MMG applications: (a) for estimating a force/MMG relation under isometric conditions and (b) for a neural network-based motion recognition, as in [30,31]. To provide a reference to surface EMG (sEMG), a measurement technique widely accepted in clinical practice, the relationship between MMG and sEMG signals recorded simultaneously during isometric measurements is shown.

## 2. Materials and Methods

All the data were collected from a single subject (male, 34 years old), who was a member of the research team, free of neuromuscular diseases and prior musculoskeletal injuries.

**Figure 1:**
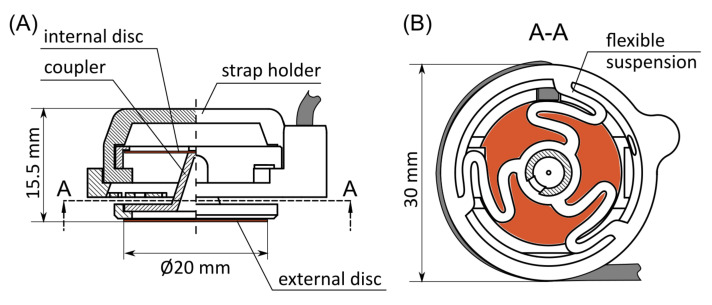
The designed mechanomyograpic (MMG) sensor: (**A**) side view and (**B**) section (A-A) through the pin of the coupler. The piezoelectric discs are marked with an orange color.

**Figure 2:**
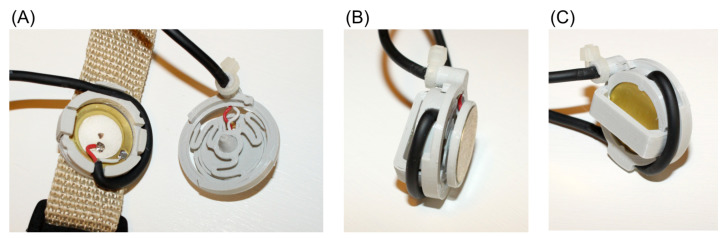
(**A**) The sensor before its final assembly. Two-core shielded microphone cables are used for signal transmission. (**B**) Side view of the assembled sensor with the external piezoelectric disc visible. (**C**) Side view of the assembled sensor with the internal disc visible.

The proposed MMG sensor consists of two piezoelectric discs in a single housing (Figure 1). The discs are excited by the MMG signal from the skin’s surface by the direct and indirect transmission of mechanical waves. The direct transmission is achieved by pressing the external disc directly to the area of interest on the skin. The indirect transmission is achieved with a coupler. The coupler consists of a base ring, which supports the external disc at its edge, and a hollow pin that contacts the central area of the internal piezoelectric disc, which is supported at four evenly distributed points. Therefore, the internal disc is working in a bending mode. The coupler is supported on flexible hinges, which allow for the sensor’s self-alignment while being attached to the subject. All the mechanical elements of the proposed sensor are 3D-printed with a PLA material (Easy PLA, Fiberlab, Brzezie, Poland) in a fused deposition modeling process. The weight of the sensor is 5.5 g without wires. The photographs of the manufactured sensor are provided in Figure 2. The dimensions of the sensor are determined mainly by the diameters of the piezoelectric discs. Due to the different mechanical interfaces between the skin and both discs, these are intended to exhibit different excitations. The resulting signal from each of them is a superposition of MMG from the acquisition site and a muscle activity coupled from the mounting strap.

The sensor was tested in two experimental setups. In the first one, the force/MMG relation under static conditions was examined with simultaneous acquisition of the sEMG signal. The second test comprised the verification of the sensor’s suitability for the classification of hand motion. Both setups employed 20 mm diameter piezoelectric discs (from unspecified manufacturer).

### 2.1. Evaluation of the Sensor Performance during Step and Ramp Isometric Contractions

All the measurements were performed under static conditions during a single session. The right arm of the subject was fixed in the measurement setup in 90${}^{{}^{\circ}}$ abduction, 0${}^{{}^{\circ}}$ flexion with 90${}^{{}^{\circ}}$ elbow joint flexion. Forearm was fixed in 0${}^{{}^{\circ}}$ pronation. The measurement setup was based on TAS606 (HT Sensors Technology, Xi’an, China) load cell. For force signal conditioning TBM4 Transbridge amplifier (WPI, Sarasota, FL, USA) was used.

The MMG sensor was placed above the middle part of the biceps brachiibelly. A surface EMG signal was acquired with a pair of wet electrodes (Kendall H92SG, Medtronic, Minneapolis, MN, USA) placed along the muscle and symmetrically with respect to the MMG sensor, 70 mm apart. Since sensor–skin interfaces of low quality are expected in real-world HMI applications, no skin preparation was performed. The reference electrode was placed in equal distance from both active electrodes (around 40 mm). Additionally, metal parts of the setup were grounded for interference noise suppression.

For signals amplification and data acquisition a custom made 8-channel MMG/EMG amplifier was used with active headstage × 100 for EMG probes and high impedance channels for the MMG transducers. For both sensors types, filtering was performed in the signal chain, specifically:

- EMG signals were high-pass (HP) filtered at the pre-amplification stage followed by an AC coupling, resulting in a 2nd order filter with 2.44 Hz cut-off frequency; the low-pass (LP) filtering was introduced with a 2nd order Sallen-Key topology with 245 Hz cut-off frequency;
- MMG signals were HP filtered with an AC coupling, which provided 1st order filter with 1.54 Hz cut-off frequency; the LP filter design was the same as for EMG signals, i.e., a 2nd order filter with 245 Hz cut-off frequency.

Signals were digitized using a 12-bit analog-to-digital converter of an STM32L476RG microcontroller (STMicroelectronics, Geneva, Switzerland), sampled at 500 sps from the range 0 ÷ 3.3 V. The digitized signals were transmitted using a serial wired connection to the computer and stored with appropriate labeling using a custom-made LabVIEW (NI, Austin, TX, USA) application.

During measurements the subject used a biofeedback provided by means of a scope (MSO2012B, Tektronix, Beaverton, OR, USA) where the force signal along with the line indicating the target force level (for step measurements) were displayed.

The target force levels were determined in relation to the maximal voluntary contraction (MVC). MVC measurements took place approximately 10 min prior to the step and ramp measurements and were performed with the same measurement setup, with the same limb position as during the following procedures. Two short (~3 s) maximal contractions, with around 3 min of rest to limit fatigue, were recorded on the scope. For each trial, a mean value of the plateau was estimated. Finally, the maximum from two measurements was considered as MVC.

The step measurements were performed for 0%, 20%, 40%, 60%, and 70% of MVC. For every step, the contraction was sustained for 30 s with around 90 s of rest between subsequent steps. In addition to the visual feedback, the subject was provided with voice information on the remaining trial time: every 5 s during the first 20 s after the required force level was reached and then every second during the last 10 s. The ramp measurements were performed by a linear increase in exerted force from 0% to 70% of MVC, which took 50 s to complete. During the ramp measurements, the voice information was initially provided every 10 s and then every second during the last 10 s. An experienced investigator visually inspected compliance of the subject’s force profiles with the respective force templates before the trials were assumed successful.

The data analysis for step and ramp isometric measurements was based on the instantaneous root mean square (RMS) values of the signals and performed using the R environment (ver. 3.4.4) [32]. For each of the acquired time series, the corresponding series of RMS values were calculated independently within subsequent 1-second long time windows (without overlapping), similarly for MMG and EMG signals. For step measurements, linear regression models were fit to the natural log-transformed values of RMS to quantify the relationships between the selected signals. The log-transformation of MMG RMS was introduced due to the reported non-linearities in the force-related MMG responses [15].

In the case of the step isometric measurements, data obtained during the experiment were used to evaluate (a) relationships between the response of each of the piezoelectric discs (internal and external) and force exerted by the muscle; (b) the relationship between responses from both of the piezoelectric discs; (c) the gain obtained at the internal disc with respect to the external disc; (d) the relationship between MMG and EMG data for both internal and external discs. Data recorded during the ramp isometric measurement were used to (a) visualize the relationship between EMG and force; (b) visualize the MMG/EMG relationship.

### 2.2. Classification Task

The piezoelectric discs were connected to charge amplifiers (custom-made amplifiers, with the feedback capacitor value set to correspond closely to the piezoelectric disc capacitance, which was measured to be 13 nF). Signals at the outputs of the amplifiers were digitized using the same setup as in the evaluation of the sensor performance during step and ramp isometric contractions.

**Figure 3:**
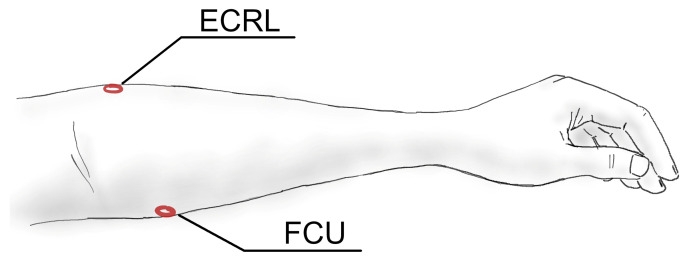
The locations of the sensors on the forearm during acquisition of the signals for the classification tasks. Labels: ECRL—externsor carpi radialis longus, FCU—flexor carpi ulnaris.

**Figure 4:**
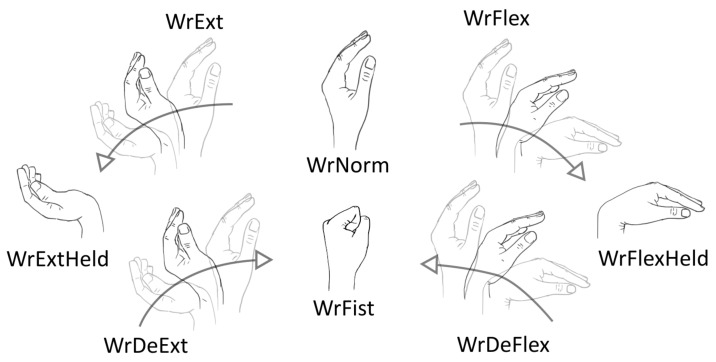
Illustration of the hand motions (positions and gestures) used in the classification tasks and their respective labels.

For the classification of hand positions and gestures, the signals were recorded from the proximal part of an unsupported forearm of a seated subject. The elbow remained bent at 90 degrees during measurements. The evaluated MMG sensor was placed above the extensor carpi radialis longus (ECRL) muscle, providing two signals: from internal (ECRL_int) and external (ECRL_ext) discs. A second MMG sensor, having a structure similar to the proposed sensor but without the external disc, was placed over the flexor carpi ulnaris muscle, thus providing a single signal from the internal disc (FCU). Both sensors shared a non-elastic, woven polyester strap that held them in place after initial tightening. The location of the sensors on the forearm is marked in Figure 3. The hand motions used in this task are listed in Table 1 and illustrated in Figure 4.

The subject was free to decide on the order of the motions executed during the measurement session. The proposed gestures are transitions between the classified positions and were included because when the online data processing is anticipated, these transitions cannot be avoided and will probably become misclassified if omitted in the training set. The time of each acquisition was fixed to 3 s, and a counting down timer was displayed to the subject to allow synchronization of the motion start with the acquisition window. The received signals were visually verified before saving to allow for discarding data if they were corrupted.

The Pearson’s correlations *r* between the signal pairs were calculated to evaluate their mutual relationships for each recording. The analysis was extended with the derived signals that had their low-frequency components additionally suppressed by filtering the raw signals in the digital domain with a 5th order Butterworth filter (5 Hz cut-off frequency). Subsequently, the distributions of Pearson’s *r* were compared visually.

**Figure 5:**
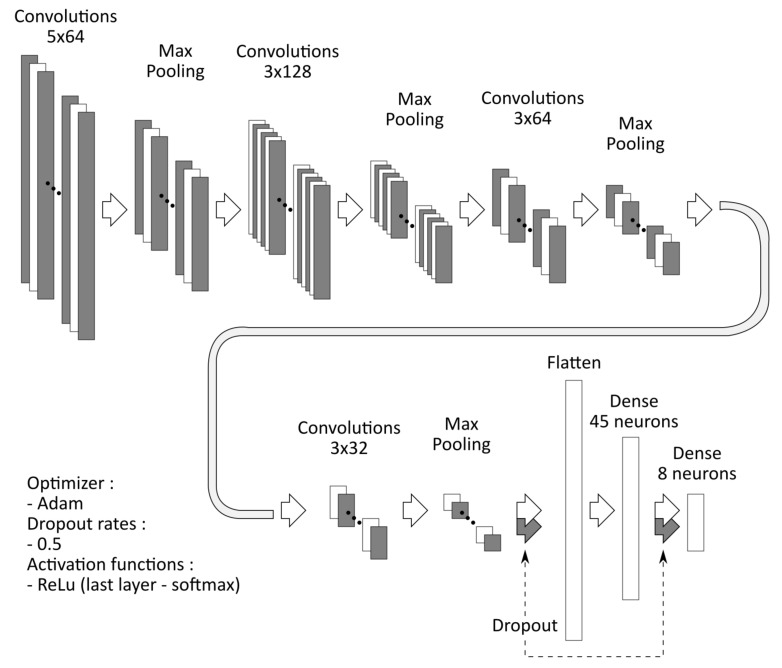
A structure of the convolutional neural network from the classification task.

The classification was performed using a 1D convolutional neural network (CNN) in the R environment (ver. 3.4.4) [32] with a TensorFlow library [33] and R interface to Keras [34]. A structure of the proposed CNN is shown in Figure 5. It consisted of four convolutional layers, each followed by a max-pooling layer. The rest of the network comprises two dense layers preceded by dropout layers (with the dropout rate set to 0.5). ReLU activation function was used throughout the internal network layers. The final layer employed a softmax activation due to the multinomial classification. The network was trained and tested using all possible combinations of signals, i.e., complete set (ECRL_int, ECRL_ext, and FCU), signal pairs (ECRL_int–ECRL_ext, ECRL_ext–FCU, and ECRL_int–FCU), and individual signals. In total, 70% of the dataset was used for training and validation. A common random seed was set for each of the combinations to ensure the repeatability of the training process. The training data were augmented using a moving window for generating three crops of 1.8 s from each original 3-s recording. A 5-fold, 5-times cross-validation was performed during training to support the choice of the number of training epochs. The final model was trained in the number of epochs which yielded the lowest value of mean loss + 1 standard error in the cross-validation phase. The overall accuracies achieved in the test set with the described CNN were calculated for each combination of signals. The confusion matrices were used for the assessment of per-class accuracies.

## 3. Results

### 3.1. Evaluation of the Sensor Performance during Step and Ramp Isometric Contractions

**Figure 6:**
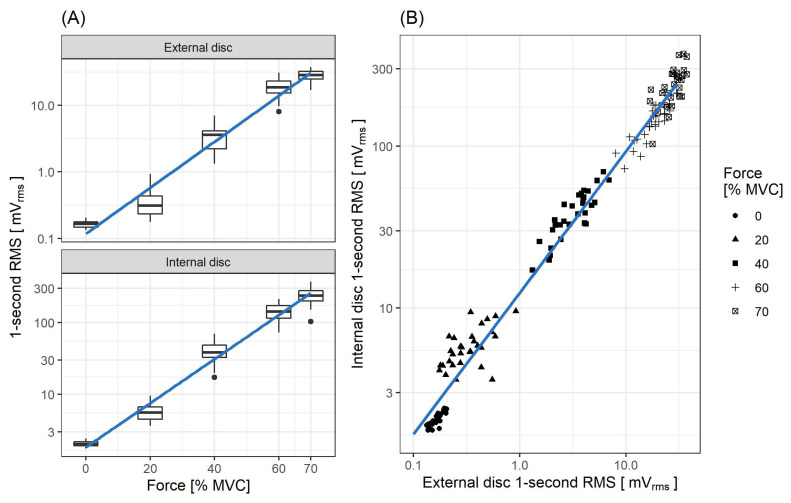
The root mean square (RMS) values of the subsequent 1-second parts (no overlapping) of MMG signals recorded during step isometric measurements. (**A**) RMS values for both of the sensor discs are plotted against the percentage of maximum voluntary contraction (MVC). The regression lines are fitted with log-transformed values of RMS. (**B**) RMS values of both signals are compared and a linear regression model in the log–log space is fitted.

The data were collected during a single session in a single trial for each of the intended contractions. Relationships between RMS and force values were modeled using the linear regression with log-transformed values of RMS (Figure 6A). The achieved $R^{2}$ coefficients were 0.953 and 0.971 for external and internal disc, respectively.

The fitted relationships, after back-transformation of RMS variable to the linear scale, are as follows:

(1) $$RMS_{ext}=0.118\;e^{0.0796\cdot F}$$

(2) $$RMS_{int}=1.82\;e^{0.0708\cdot F}$$

where: *F* is the force given in [% MVC], $RMS_{int}$ and $RMS_{ext}$ are the RMS values of signals recorded from the internal and external piezoelectric discs, respectively, both given in [mV].

To estimate dependency between RMS values obtained for internal and external discs, a linear regression model was fitted ($R^{2}=0.983$) to the log–log transformed data (Figure 6B). Back-transformation of the model yields the relationship:

(3) $$RMS_{int}=12.4\;RMS_{ext}^{0.873}$$

**Figure 7:**
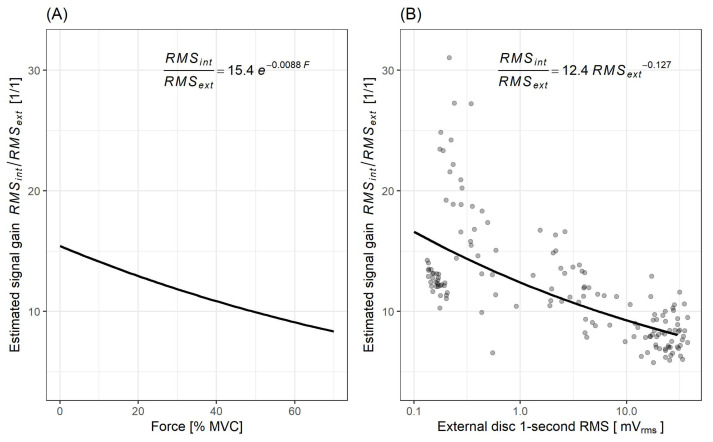
Estimated signal gain ($RMS_{int}/RMS_{ext}$) as a function of: (**A**) force (*F*), represented as % MVC, (**B**) $RMS_{ext}$ (data-points recalculated based on experimental data).

Based on Equations (1) and (2) the signal gain for F∈[0,70] and $RMS_{ext}\in \;\left[0.1,30\right]$ was estimated as $RMS_{int}/RMS_{ext}$. The calculated gain/force relation is presented in Figure 7A. Similarly, based on Equation (3) the gain as a function of $RMS_{ext}$ was calculated and presented in the Figure 7B, along with recalculated data-points.

**Figure 8:**
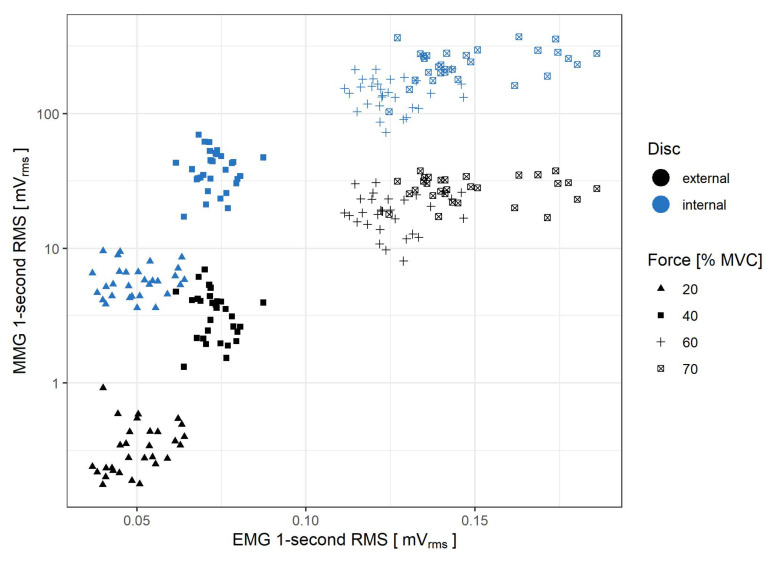
Comparison of MMG and EMG RMS values obtained during step isometric contraction measurement. The 0% MVC level is omitted, as it does not reflect muscle activity. The comparison is made for both internal (blue) and external (black) piezoelectric discs.

In Figure 8 the relationship between EMG and MMG recorded from both external and internal piezoelectric discs during step isometric contractions is shown.

**Figure 9:**
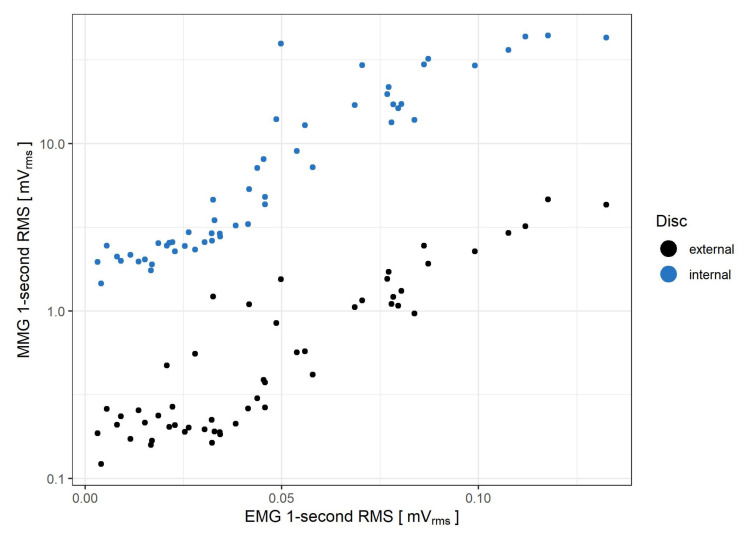
Comparison of MMG and EMG RMS values obtained during the ramp isometric contraction measurement. The comparison is made for both internal (blue) and external (black) piezoelectric discs.

In Figure 9 the relationship between EMG and MMG recorded from both external and internal piezoelectric discs during ramp isometric contraction is shown.

**Figure 10:**
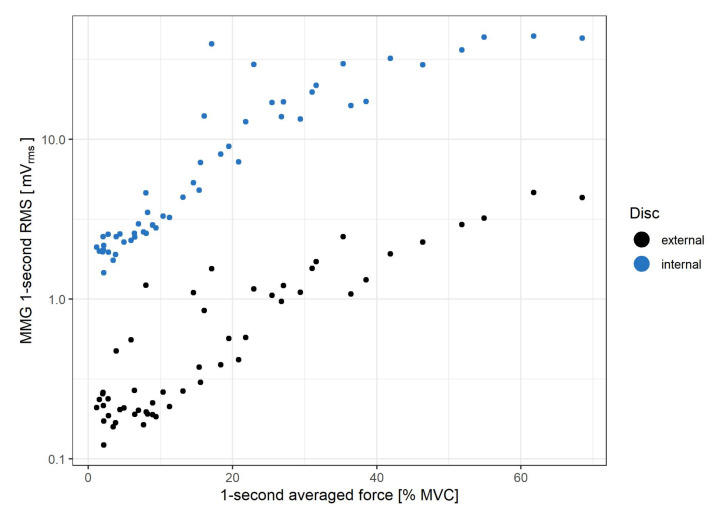
MMG as function of force exerted by the muscle during the ramp (from 0% to 70% MVC in 50 s) isometric contraction. Data are plotted both for internal (blue) and external (black) piezoelectric discs.

Figure 10 illustrates the relationship between MMG and force signals obtained for the ramp isometric measurement.

### 3.2. Classification Task

**Figure 11:**
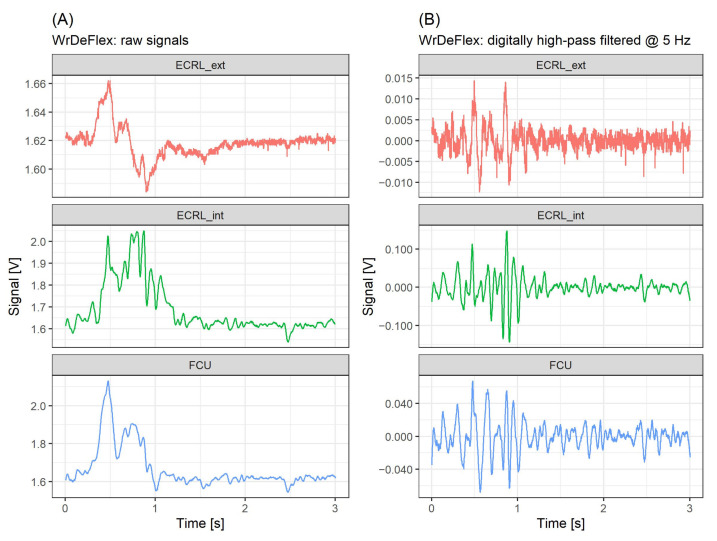
Example of signals acquired during the wrist de-flexion (WrDeFlex): (**A**) raw and (**B**) digitally high-pass filtered at 5 Hz. The signal sites abbreviations: ECRL_ext\ECRL_int–extensor carpi radialis longus, external\internal piezoelectric disc of the sensor, respectively; FCU—flexor carpi ulnaris, internal piezoelectric disc of the sensor.

The data were collected during two sessions. For each classified motion, 35 repetitions were acquired, yielding in total 280 records. In each session, the measurements were taken from the left forearm. An example of signals acquired during the wrist de-flexion (WrDeFlex) is provided in Figure 11.

**Figure 12:**
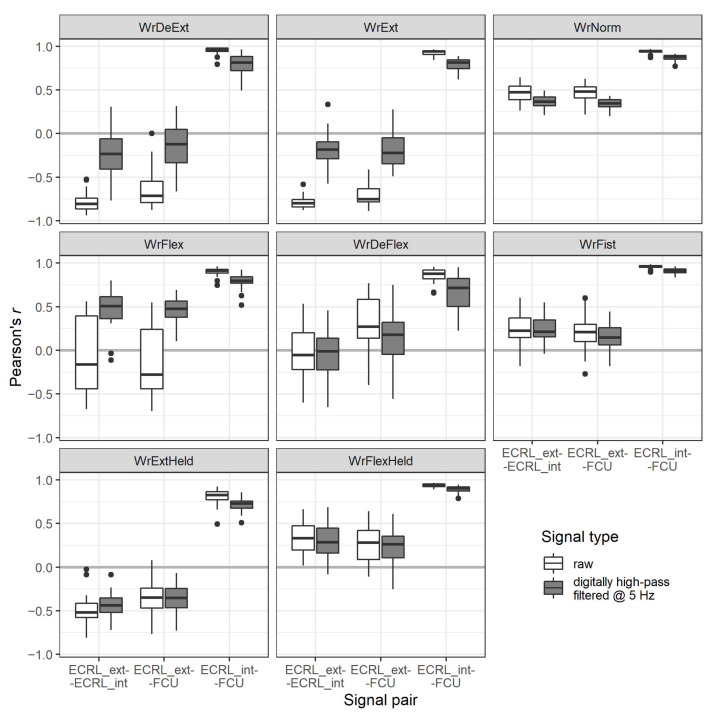
The Pearson’s correlations between the signals, grouped by the type of executed motion (see Table 1 for the motion labels description and Figure 11 for signal sites abbreviations). The correlations are given for raw and digitally high-pass filtered signals with a 5 Hz cut-off frequency.

The Pearson’s correlations *r* between the signal pairs were calculated for each recording to evaluate their mutual relationships. The results for both raw and digitally filtered signals are shown in Figure 12.

For all motions, the correlations between the raw signals from the internal disc of the sensor placed above the ECRL and the internal disc of the sensor placed above the FCU (i.e., ECRL_int–FCU pair) are visibly high, and for most cases, *r* is between 0.8 and 1.0. The remaining two pairs of signals show relatively lower absolute correlations. Only in the case of gestures WrDeExt and WrExt their absolute *r* values approach these of the pair ECRL_int–FCU. However, for these gestures, correlations between the ECRL_int–ECRL_ext, and ECRL_ext–FCU pairs are negative.

For the digitally high-pass filtered signals, when compared to the raw signals, the main observed differences involve correlations between the ECRL_int–ECRL_ext, and ECRL_ext–FCU pairs for:

- WrDeExt and WrExt gestures, where absolute *r* values are diminished significantly;
- WrFlex gesture, where the distributions of *r* values are narrowed and shifted to the positive values.

For all motions, the ECRL_int–FCU pair correlations are slightly diminished but remain positive and relatively high for the filtered signals.

**Figure 13:**
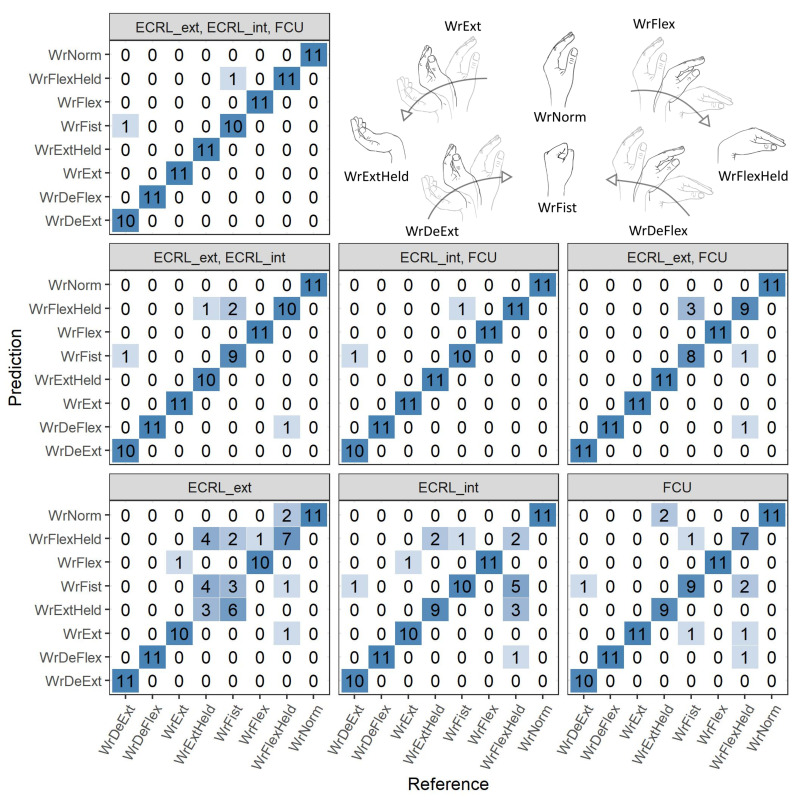
Confusion matrices for all trained models, headers of matrices indicate the signal set used (see Table 1 for the description of the motion labels and Figure 11 for the description of the signal labels). Illustrations of classified hand motions are recalled for convenience.

The accuracies achieved with the described CNN in the test set are presented in Table 2. Confusion matrices for all trained models are provided in Figure 13.

## 4. Discussion

### 4.1. Evaluation of the Sensor Performance during Step and Ramp Isometric Contractions

The MMG RMS (log-transformed) for both piezoelectric discs of the sensor exhibits a marked correlation with the force exerted by the muscle during step contractions, as observed for up to 70% MVC: $R^{2}$ coefficient equals 0.953 and 0.971 for the external and internal disc, respectively. However, as the measured force exceeds 60% MVC, the rate of the MMG RMS increase begins to decline for both the step and ramp measurements. Such a change is in line with findings from previous studies of the biceps brachiimuscle isometric force generation, where plateaus or decreases of MMG amplitude were observed from approximately 60–80% MVC [10,35]. An interesting feature of the presented sensor is that for force levels below 20% MVC, the rate of MMG RMS increase is lower for the external disc than the internal one. This occurs likewise in step (Figure 6A) and ramp (Figure 10) contractions. Moreover, it has been previously documented that the MMG response to the force varies with the muscle fiber type composition and, for the ramp contractions, also depends on the type of muscle action (increase vs. decrease) [36]. Therefore, the sensor’s usage in HMI applications exploiting the force/MMG relationship must account for differences in muscle composition, type of contraction (stable vs. ramp) and may be limited to the force values not exceeding 60% MVC, with additional preference for the internal disc signal if the force remains below 20% MVC. The observed MMG/sEMG relationship shows similar MMG RMS saturation from approximately 60% MVC, contrary to the sEMG RMS, which continues to rise with the exerted force (Figure 8 and Figure 9).

Looking into the sensor-related details, MMG signal obtained from the internal disc has a considerably higher amplitude than the one acquired from the external disc. This is a feature that is beneficial for the design of signal chains, where it enables, for example, a replacement of the electronic amplification of a signal by a mechanical one, or, when combining measurements from two sensors having different gains and placed at a single site (as in the coupled sensor presented here), it extends the covered dynamic range. However, the achieved gain changes with the exerted force level from around 15 V/V for minimal force to around 8 V/V for 70% MVC. Nevertheless, one could observe a high correlation ($R^{2}$ = 0.983) between log–log transformed RMS of MMG signals from both piezoelectric discs. This should be expected, as both transducers are mechanically coupled. Thus, the advantage of having both discs at a single site is not obvious (beyond the mentioned possibility of extending the dynamic range), at least for operation under static conditions, when the signal’s RMS is used. However, they should not be considered redundant for all static applications, as the back-transformation of the regression equation indicates a non-linear relationship (Equation (3)) between both signals. Depending on the signal processing method chosen, the mutual non-linearity at the system input may provide benefits when signals are superimposed.

### 4.2. Classification Task

The best-performing models in the classification task yielded accuracies in a range from 94.3% to 97.7%, similar to those reported in referenced works: up to 96% [31] and from 85.6% to 95.1% [30]. However, the direct comparison of results was not carried out due to differences in the sets of considered positions and gestures. In the presented setup, the gestures had the highest classification accuracies for all signal combinations, while hand positions were more often misclassified. The performance of CNN models was visibly lower for the 1-signal inputs with the rise in the misclassification rate for the following positions: flexed and extended wrist, clenched fist. The mentioned hand positions are inevitably more challenging to classify than gestures, as the latter yield significantly higher signal levels that result from the large change of muscle shape during the onset of the motion.

Analysis of the correlations of filtered and raw signals showed that when gestures were performed, a significant, low-frequency component below 5 Hz was present, which differentiated ECRL_ext from the rest of acquired raw signals, as shown in Figure 12. Thus, unlike for measurements made for isometric contractions, where combining signals from a single site (ECRL_ext and ECRL_int) should be possible and potentially beneficial, here it would result in a loss of information, and the usability of such a derived signal may vary depending on the characteristics of the considered gestures. However, signals from the ECRL_int and FCU sites were highly correlated when acquired during all chosen hand motions, which indicates their possible interchangeability. Results of the classification task also supported this conclusion, as the models that were based on the combinations: ECRL_ext–ECRL_int (single-site, 2-signal configuration) and ECRL_ext–FCU (double-site, 2-signal configuration), provided similar overall test accuracies. At the same time, the models based on double-site configurations (i.e., yielding 2- and 3-signal sets) provided accuracies better or equal to the single-site, 2-signal one. The single-site, 1-signal configurations resulted in the lowest classification accuracies.

We have shown that attaching the sensor to the examined limb with a strap allows for transmitting usable signals from the encompassed area to the sensor’s internal piezoelectric disc. In other words, a source of potential crosstalk has been turned into a valid input to the classification algorithm. Therefore, the proposed coupled single-site sensor may be considered a replacement for the two-site arrangement. As a result, it may simplify the myographic interface in terms of sensors’ spatial distribution. In our proof of concept, the substitution of the two-site sensor pair with a single-site sensor resulted only in a slight reduction in the classification accuracy (i.e., it decreased from 97.7% to 94.3%).

### 4.3. Observations on the Sensor Application

Based on the review of the acquired data, a few points regarding the sensor operation should be highlighted. These are general remarks that may be helpful when it comes to the implementation of the sensor in the MMG interface. The presented sensor design with a coupling pin that is free to move relative to the internal disc sporadically led to artifact generation due to the short-range repositioning of the pin contact point during large muscle motion. Such a movement resembles a stick-slip phenomenon and is observed as a narrow pulse, simultaneously in signals from both discs. However, these pulses occurred infrequently and likely did not pose noticeable issues during classification and did not introduce significant RMS analysis biases. The cardiac interference was the second source of a non-myographic signal, most apparent in the data recorded for stable hand positions. It may be treated as an auxiliary input for some applications and employed, e.g., for heart rate estimation. The next observed property stems from the difference in disc gains. Due to the higher gain achieved for the internal disc, its signal chain was prone to saturation when gestures were executed rapidly. Eventually, these had to be performed with a moderate speed, and acquisitions with saturation were discarded. However, the signal from the external disc had rarely saturated during faster motions. Even in such events, the sensor as a whole continued to provide a partially valid output. This feature may be beneficial for applications that involve both slow and fast movements, as the signal saturation may be easily detected and eventually managed in an algorithm or research protocol.

Another valuable feature of the designed sensor is that it may be manufactured from easily accessible and low-cost materials, and its assembly is not a demanding process. The 3D-printed parts required approximately 3000 mm${}^{2}$ of a PLA material (1.22 m of 1.75 mm filament), with the cost of manufacturing (including electric energy and material) estimated as EUR 0.2. The price of a single piezo disc was approximately EUR 0.2. Without the wire and an optional connector, the cost of components of the proposed sensor is approximately EUR 0.6.

### 4.4. Limitations of the Study

As the data were collected from a single subject, no evaluation of an inter-subject variability of the sensor’s performance was possible. Moreover, collecting more measurements from the same subject over an extended period would be beneficial to establish repeatability characteristics of the sensor. In this study, the subject was additionally a research team member. Therefore, he may be considered a skilled user of the developed sensor, which may introduce a bias resulting in increased performance if compared with a person having no or limited prior experience with similar instrumentation. Even though omitted in the proof of concept presented here, such analyses would be of significant importance for every specific application of the coupled MMG sensor, where more participants having different skill levels in terms of using the designed interface should be recruited.

## 5. Conclusions

We have confirmed that the simplification of the MMG interface in terms of transducers’ spatial arrangement is possible with the designed sensor. Results of its evaluation under static and dynamic conditions support its potential for application in HMI.

Further investigation is desirable to identify the source of the differences between the vibration transmission to the internal and external disc. These differences seem to arise not only from the sensor’s mechanical structure but to a large extent from the sensor-skin interface. A partially related issue of adipose tissue influence on MMG was discussed, e.g., by Santos et al. [18]. In subsequent studies aimed at refining the requirements of the application of the proposed sensor, one should consider measuring the skinfold thickness or the subcutaneous adipose tissue to characterize the application site better. In general, it would be beneficial to obtain a model that would make it possible to optimize the sensor characteristics depending on the application requirements. Furthermore, the sensor’s performance should be tested with more muscle groups (which exhibit different recruitment of motor units and firing rates) and muscle actions (e.g., isokinetic contractions). Lastly, introducing a second myographic modality while preserving the proposed mechanical structure is worth considering, like in a work by Ke et al. [37], where a hybrid EMG-FMG sensor for prosthetic control was presented. For example, an sEMG–MMG integration may significantly reduce the EMG interelectrode distance, which had to be relatively large in our study due to the MMG sensor size.

## Figures and Tables

**Table 1:** The hand motions (positions and gestures) used in the classification tasks and their respective labels.

Motion	Label
Positions:	
Normal wrist and hand position	WrNorm
Extended wrist	WrExtHeld
Flexed wrist	WrFlexHeld
Clenched fist	WrFist
Gestures:	
From normal position to extended wrist	WrExt
From extended wrist to normal position	WrDeExt
From normal position to a flexed wrist	WrFlex
From flexed wrist to normal position	WrDeFlex

**Table 2:** Classification task results—test accuracies (see Figure 11 for the description of the signal abbreviations).

Signal Set	Cross-Validated Training Epochs	Overall Test Accuracy
ECRL_ext, ECRL_int, FCU	35	97.7%
ECRL_ext, ECRL_int	35	94.3%
ECRL_int, FCU	38	97.7%
ECRL_ext, FCU	35	94.3%
ECRL_ext	24	75.0%
ECRL_int	33	84.1%
FCU	39	89.8%

## Data Availability

The data presented in this study are available on request from the corresponding author.
